# Prognostic Value of Serum Biomarkers in Patients with Idiopathic Pulmonary Fibrosis in Relation to Disease Progression

**DOI:** 10.3390/jpm13091307

**Published:** 2023-08-26

**Authors:** Kalliopi Domvri, Ioannis Organtzis, Apostolos Apostolopoulos, Evangelia Fouka, Theodoros Kontakiotis, Despoina Papakosta

**Affiliations:** 1 Lung Immunology and Bronchoalveolar Lavage Unit, Pulmonary Department, Medical School, Aristotle University of Thessaloniki, George Papanikolaou Hospital, 57010 Thessaloniki, Greece; aposapos92@gmail.com (A.A.); depapako@gmail.com (D.P.); 2 Out-Patient Clinic for ILDs, Pulmonary Department, Medical School, Aristotle University of Thessaloniki, George Papanikolaou Hospital, 57010 Thessaloniki, Greece; giannisorgantzis@yahoo.com (I.O.); evafouka@gmail.com (E.F.); kontak@auth.gr (T.K.)

**Keywords:** biomarkers, prognostic, lung, fibrosis, serum

## Abstract

Background: The aim of this present study was to determine serum biomarker levels and their correlation with respiratory function and the clinical course of patients with idiopathic pulmonary fibrosis (IPF). Materials and Methods: This study included 72 IPF patients, according to the ATS/ERS criteria, in whom antifibrotic treatment was initiated. Blood samples were taken, and serum biomarkers, such as KL-6, SP-D, CCL18, CXCL13, VEGF-A, IL-8, IGFBP-1, IGFBP-2, IGFBP-7 and ICAM-1 were measured using ELISA methodology. Pulmonary function tests (FVC, TLC, DLCO-% pred) were determined at baseline and after 12 and 24 months and analyzed in correlation with the biomarkers. Results: The majority of patients (mean age 72 ± 6 years) were men (83%). The FVC and DLCO values at the 12-month follow-up were found to be statistically decreased in deceased patients (*p* < 0.05). The SP-D (*p* < 0.001) and the IGFBP-1 (*p* = 0.021) levels were found to be increased at the 1-year follow-up in deceased patients, and similarly, the SP-D (*p* = 0.005) and ICAM-1 (*p* = 0.043) levels at the 2-year follow-up. A chi-square test revealed that 70% of the category IV GAP index was found with cut-off elevated levels of a biomarker combination (KL-6, SP-D, VEGF-A) from the ROC curve analysis (*p* < 0.05). Conclusion: This study provides evidence, for the first time in a Greek population, of the possibility of using a combination of KL-6, SP-D, and VEGF-A serum levels along with the GAP index.

## 1. Introduction

Idiopathic pulmonary fibrosis (IPF) is a chronic and irreversible fatal interstitial lung disease of unknown etiology [1]. The clinical course is highly heterogeneous, although limited to the lungs, and is characterized by damage to lung tissue by inflammation and fibrosis compatible with the histology of usual interstitial pneumonia. To date, there is no curative treatment and only two biological agents, pirfenidone and nintedanib, have been approved by the FDA as a standard of care. Both drugs have been shown to slow the progression of IPF in randomized clinical trials [2,3,4]. However, there is great variability in the clinical course of IPF patients, including long periods of stability, a steady, gradual decline, and/or periods of acute deterioration. Recently, studies highlighted the increasing impact of genetics on aspects related to pathophysiology, accurate and early diagnosis, and the treatment and prevention of IPF, though this requires further investigation [5,6,7]. Thus, predictors of survival in everyday clinical practice are needed for physicians and patients.

It has been known for a long time that transforming growth factor beta (ΤGF-β) is involved in the pathophysiology of IPF. However, the pathogenesis of IPF progression is still unclear regarding whether it is related to epithelial injury from endogenous or exogenous events, which results in widespread fibrosis, replacing the normal lung parenchyma [1]. Key cells in the pro-fibrotic process include myofibroblasts, alveolar epithelial cells, fibroblasts, immune cells and endothelial cells. Through the “epithelial-mesenchymal transition”, several other biomarkers are implicated in the outcome of the disease [8,9]. Most of these factors belong to chemokines, such as C-C motif ligand 18 (CCL18) and interleukin-18 (ΙL-18), matrix metalloproteinases (MMP-1, MMP-7 and MMP-9) and growth factor families, such as immunoglobulin G-binding proteins (IGBP-1, IGBP-2 and IGFBP-7). Other biologic markers in the alveolar epithelial cells include the Krebs von den Lungen-6 (KL-6) antigen and the surfactant proteins A and D (SP-A, SP-D). Additional markers that serve as potential diagnostic or prognostic tools concerning endothelial damage are vascular endothelial growth factor (VEGF), intercellular adhesion molecule 1 (ICAM-1) and other fibrogenesis and extracellular remodeling markers, such as myeloperoxidase (MPO).

Recent research has suggested that the S100 calcium-binding protein A12 (S100A12) could potentially serve as a prognostic serum biomarker in IPF [10]. Furthermore, according to studies, genomic approaches may include the identification of microRNAs and the polymorphism in the promoter region of MUC5B, as well as some other rare mutations [11,12]. Regarding novel approaches, recently, the serum proteomic profile has the potential to offer valuable insights into the heterogeneity of IPF and to uncover protein alterations that can aid in its diagnosis and treatment decisions [13]. Also, a different approach for risk prediction and survival of IPF is the use of the GAP index (gender, age and pulmonary function) [11]. Moreover, a recently published study suggested that the GAP index combined with the Charlson Comorbidity Index (CCI) could provide more accurate information for predicting prognoses in patients with ILD when compared to the GAP index scoring system alone [14].

Although biomarkers alone have shown little success as diagnostic or prognostic tools, when taken together, according to studies, changes in blood proteins or cells could predict disease progression [15]. Furthermore, recognizing a combination of biomarkers that characterize a subset of patients could help physicians choose better patients to participate in clinical trials.

To our knowledge, this is the first study investigating a wide range of biomarkers in relation to the clinical course and GAP index in IPF patients. The purpose of this study was to explore the prognostic value of serum biomarker levels for disease assessment and survival.

## 2. Materials and Methods

### 2.1. Patients

This is a prospective cohort study that included 72 patients with idiopathic pulmonary fibrosis, diagnosed according to the American Thoracic Society (ATS)/European Respiratory Society (ERS) criteria [16,17]. Specifically, the diagnosis was based on clinical evaluation, confirmed according to the criteria of high-resolution computed tomography and laboratory findings to exclude connective tissue diseases [17,18]. The study protocol was approved by the Local Ethics Committee of George Papanikolaou Hospital (317, 15 June 2019). All participants provided informed consent to participate in the study. The patients were followed up in the out-patient clinic for ILDs, in the Pulmonary Department, Aristotle University of Thessaloniki, for 2 years, from June 2019 to December 2021, after having started antifibrotic treatment (nintedanib or pirfenidone) as the standard of care.

### 2.2. Pulmonary Function Tests

Pulmonary function tests (FVC, TLC, DLCO-% pred) were determined at 3 time points (initial value, 12 and 24 months). The patients were monitored for 24 months after their serum sample collection. The variables included the first second of forced expiration (FEV1), FEV1% predicted, forced vital capacity (FVC), FVC% predicted, total lung capacity (TLC), TLC% predicted, diffusing capacity of the lung for carbon monoxide (DLCO), and DLCO% predicted, which was determined using the JAEGER Masterscreen PFT system, provided that they were in a stable condition. According to hospital policies, a diagnostic PCR-based test was performed to exclude SARS-CoV-2 infection before spirometry. Also, the gender–age–physiology (GAP) index was calculated for each patient (gender, age, FVC and DLCO) [19], and the objective burden of comorbidities was determined using the Charlson Comorbidity Index (CCI) [20].

### 2.3. Blood Collection

Blood samples were collected from participants, provided that they were in a stable condition or without any recent inconvenience and without any antifibrotic treatment as a standard of care before the initiation of the study. Then, blood was kept at room temperature for one to two hours to be clotted and then centrifuged for 10 min, and the serum was extracted. Serum samples were frozen at −80 °C. 

### 2.4. Measurement of Serum Biomarkers

Thirteen serum biomarkers, such as KL-6, SP-D, CCL18, CXCL13, VEGF-A, IL-8, IGFBP-1, IGFBP-2, IGFBP-7, MMP-1, MMP-9, MPO and ICAM-1, were determined using panel kits (AimPlex Biosciences) tested by flow cytometer analysis using a BD FACSCalibur system (BD Biosciences, San Jose, CA), according to the manufacturer’s recommendations and instructions. Intra-assay and inter-assay variabilities of the serum cytokine measurements were CV:<10% and CV:<20%, respectively. Specifically, 45 μL of the serum sample and 45 μL beads of the panel kits were mixed and then incubated for 1 h at room temperature. After incubation, 0.5 mL of wash buffer was added, and the samples were centrifuged for 5 min. Samples were incubated first with a biotin-conjugated monoclonal antibody (30 min) and then subsequently incubated with streptavidin-conjugated monoclonal antibody (20 min). Finally, a wash reading buffer was added to all samples. The data were evaluated using FlowJo software (ver. 7.6; TreeStar Inc., San Carlos, CA, USA). KL-6 and SP-D proteins were measured by commercially available ELISA assay kits, according to the manufacturer’s protocols, Antibodies-online GMBH and Biotechne, and the R and D Systems, respectively. 

### 2.5. Statistical Analysis

Statistical analysis was performed using SPSS 21.0 software (SPSS Inc., Chicago, IL, USA). Comparing numerical data between groups was performed with a t-test, and the χ2 test was performed for the categorical variables. As with the nonparametric data, the Mann–Whitney U test was used to assess differences between the IPF group of patients. A paired t-test or Wilcoxon test for the nonparametric values was used to assess the differences of the same parameter at different time points. The correlation parameters were obtained using Pearson’s correlation coefficient (r). Receiver operating characteristic (ROC) analyses were performed for the mathematical expression of distinct serum biomarker concentrations as cut-off points. The Kaplan–Meier curve with a log-rank test was used for survival analyses. All data were expressed as the mean ± standard deviation (SD), and *p-* values of <0.05 were considered statistically significant. 

## 3. Results

### 3.1. Demographic and Clinical Characteristics of the Study Patients

In total, 72 patients were included in the study, with an average age of 72 ± 6 years, and 83% were of male predominance. The demographic and treatment characteristics of the subjects are given in Table 1. Older and male patients were statistically significantly found in the group of deceased IPF patients (*p* < 0.05). Most of the participants were current or heavy ex-smokers (mean of 77.2 packs/year). There was a statistically significant use of long-term oxygen therapy (LTOT) and corticosteroids among the group of IPF patients who were deceased at 24 months of follow-up. Regarding the Gender–Age–Physiology (GAP) index, deceased patients, after 2 years, were categorized in group IV (*p* < 0.05).

The pulmonary function results at different time points are presented in Table 2. The FVC and DLCO values at the 12-month follow-up were found to be statistically decreased in deceased patients (*p* < 0.05). As patients were previously diagnosed, data related to the disease’s onset were not included. 

### 3.2. Relationship between Serum Biomarker Levels and Survival

The mean values of the serum biomarker levels in IPF patients appear in Table 3. Concerning the 12-month follow-up, SP-D was significantly increased in deceased patients at the one-year follow-up (35 ± 25 → 60 ± 17 ng/mL, *p* < 0.001) and the IGFBP-1 values were significantly decreased (411 ± 75 → 802 ± 141, *p* = 0.021). The SP-D and ICAM-1 levels were found to be significantly higher in patients who were deceased at the two-year follow-up (*p* < 0.05). 

**Figure 1:**
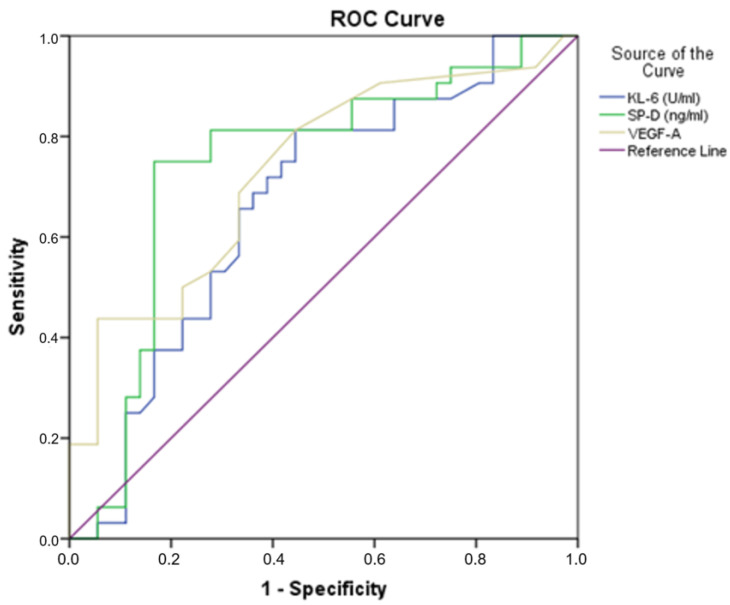
Receiver operating characteristic (ROC) curve analyses of KL-6, SP-D and VEGF-A, distinguishing IPF patients that were deceased at 24 months.

Receiver operating characteristic (ROC) curve analysis was performed to test if the baseline serum values were predictive of the survival of IPF patients. The ROC curve analysis showed serum levels of KL-6, SP-D and VEGF-A to be predictive of survival in our cohort of IPF patients (Figure 1), with a highly statistically significant relationship (Table 4). The ability of each biomarker to predict disease severity was estimated by measuring the area under the receiver operating characteristic curve (AUC). Furthermore, to predict survival with the combination of KL-6, SP-D and VEGF-A levels—either two out of three or all three biomarkers—we performed a chi-square test above the cut-off points, as reported in Table 4. A combination use of these three biomarkers could predict survival at the 12- and 24-month follow-up (*p* = 0.001). The presence of these biomarkers did not reveal any more associations with other demographics or clinical characteristics. Regarding smoking status, no significant associations were revealed in relation to the survival statuses at the 12- or 24-month follow-up and ROC curve analysis.

### 3.3. Relationship between Gender and Lung Function with Biomarker Serum Levels

**Figure 2:**
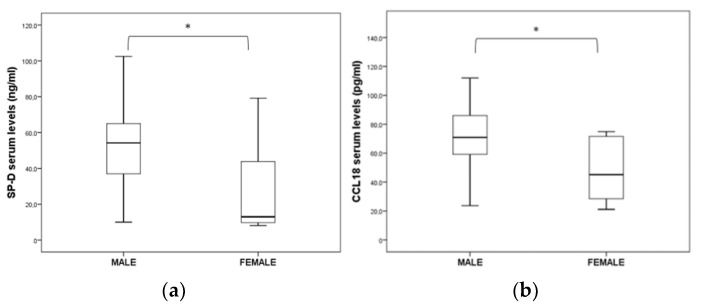
Significant differences in (**a**) SP-D and (**b**) CCL18 serum levels between male and female IPF patients (* *p* < 0.05).

Concerning gender distribution, the SP-D (*p* = 0.001) and CCL18 (*p* = 0.009) serum levels were found to be statistically increased in male IPF patients when compared to females (Figure 2). By contrast, there was no significant difference between the genders regarding the levels of KL-6, CCL18, CXCL13, IL-8, IGFBP-1, IGFBP-2, MMP-1, MMP-9, MPO and ICAM-1.

In this study, in terms of lung function, no statistical differences were found among all IPF patients with more than a 10% change in the FVC and TLC predicted values with serum biomarker levels. In deceased IPF patients with more than a 10% change in the FVC value, SP-D levels were found to be significantly higher (*p* < 0.05). Furthermore, we found an inverse relationship between the SP-D and spirometry values of DLCO% predicted in deceased subjects at 12 (*r* = −0.582, *p* = 0.001) and 24 (*r* = −0.642, *p* = 0.013) months of follow-up. Other inverse correlations between the DLCO % predicted values at 24-month follow-up were found with IGFP-1 (*r* = −0.600, *p* = 0.020) and 2 (*r* = −0.600, *p* = 0.039) and IL-8 (*r* = −0.591, *p* = 0.026), and similarly, between the TLC % predicted values with VEGF-A (r = −0.724, *p* = 0.008), KL-6 (*r* = −0.775, *p* = 0.003), CCL18 (r = −0.617, *p* = 0.033) and CXCL13 (*r* = −0.675, *p* = 0.016) at the 24-month follow-up. This was also seen between the FVC % predicted values with MPO (*r* = −0.552, *p* = 0.041), IGFBP-7 (*r* = −0.537, *p* = 0.032) and CXCL13 (*r* = −0.547, *p* = 0.028) at 12 months and the FVC% predicted values with MPO (r = −0.577, *p* = 0.049), IGFPB-1 (*r* = −0.672, *p* = 0.017), IGFBP-7 (*r* = −0.559, *p* = 0.038) and CXCL13 (*r* = −0.551, *p* = 0.041) at the 24-month follow-up.

### 3.4. GAP Index Relation to Survival and Serum Levels

The GAP index was significantly related to mortality at the 12- and 24-month follow-ups (Table 5). Moreover, a chi-square test revealed that 70% (*n* = 14) of the category IV GAP index were found with cut-off elevated levels of the biomarker combination (KL-6, SP-D, VEGF-A) from the ROC curve analysis (*p* < 0.05).

### 3.5. Charlson Comorbidity Index Relation to Survival, Serum Levels and GAP Index

According to the CCI, most of the patients were categorized in groups 2–3 (Table 6) and similarly, after the 24-month follow-up (*p* < 0.05). Moreover, a chi-square test revealed that 80% (*n* = 16) of category IV of the GAP index were found in groups 2–3 of the CCI (*p* < 0.05). Our analysis revealed no further associations.

### 3.6. Comparing Antifibrotic Treatment and Serum Biomarker Levels

**Figure 3:**
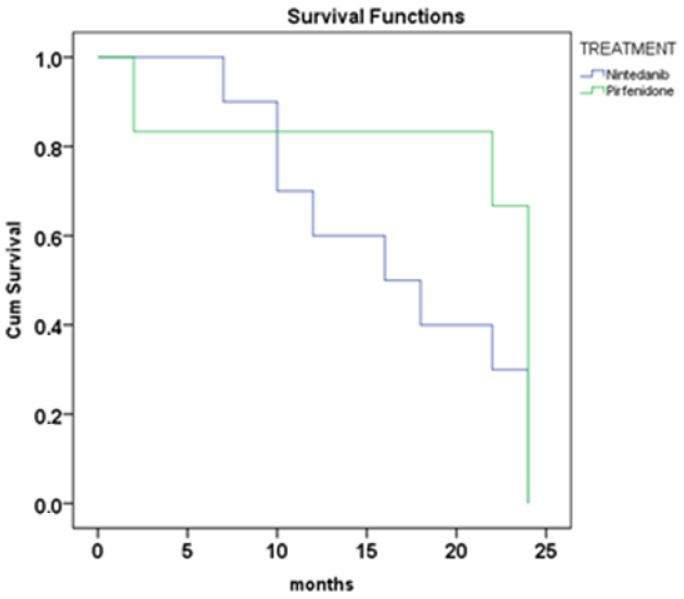
Survival analysis Kaplan–Meyer at 24 months of treatment with nintedanib and pirfenidone (log-rank, *p* = 0.054).

The relationship between antifibrotic treatment and the serum levels of biomarkers was investigated. The survival analysis showed no significant differences between nintedanib and pirfenidone treatment in our patients (Figure 3) (*p* = 0.054, 16 vs. 24 months, median survival).

## 4. Discussion

IPF is a highly complex disease. Currently, approved drugs have not succeeded in reversing the fibrotic process or even stabilizing lung function [21]. Moreover, there are no biomarkers that could stratify the IPF patients by predicting disease severity or responsiveness to treatment [22]. In this study, we investigated an important number of potential biomarkers to predict survival in IPF patients at the time of initiation of their antifibrotic treatment. The most important finding of our study was that the combination of elevated levels of KL-6, SP-D and VEGF-A, along with the GAP index, was associated with the worst disease clinical course and survival. Moreover, the IGFBP-1, ICAM-1 and CCL18 serum levels were significantly elevated in IPF patients with disease progression and death.

More specifically, in our study, we used ROC analysis to establish the cut-off values for the KL-6, SP-D and VEGF-A concentrations as indicators of survival in our cohort of IPF patients. Using the cut-off values revealed by the ROC curve and after performing the chi-square test, the use of the combination of these three biomarkers resulted in the prediction of survival at 12 and 24 months. Subjects with values above the cut-off points were significantly correlated to mortality. The presence of these biomarkers did not show any more associations with other demographics or clinical characteristics. 

Recently, several efforts have been made towards the identification of accurate predictors. KL-6, a high molecular weight glycoprotein, is expressed in type II pneumocytes and bronchiolar epithelial cells [23]. Studies have shown that serum levels of KL-6 correlate with the extent of lung fibrosis and disease progression in IPF patients [24]. Elevated levels of KL-6 may indicate the presence and severity of lung damage, as well as alveolar-capillary permeability [25]. Although, in our study, no statistically significant differences were found in the KL-6 levels between deceased and alive patients at a one or two-year follow-up. Patients with KL-6 serum values of >607 U/mL were associated with worse survival, whereas Wakamatsu et al. concluded that patients with initial serum KL-6 values of >1000 U/mL had worse prognoses than those with <1000 U/mL [26]. In Japan, KL-6 has been approved as a diagnostic biomarker for interstitial lung diseases since 1999, and it has been used in clinical practice to help diagnose and monitor patients with IPF [23]. Furthermore, elevated levels of SP-D in the blood or bronchoalveolar lavage fluid have been associated with the severity of lung fibrosis and disease progression in IPF patients [27]. In a meta-analysis of 21 papers totaling 1289 IPF patients, researchers concluded that serum SP-A/D detection might be useful for differential diagnosis and prediction of survival in those patients [28]. VEGF-A has also been implicated in the development of IPF, as it stimulates the proliferation and migration of fibroblasts, which may contribute to the development of lung fibrosis [29]. Although VEGF-A has been reported to have inconsistent results [30], like in our study, among several serum biomarkers studied, researchers found that VEGF had the largest AUC for predicting disease severity [30].

Physiologic parameters, such as an FVC change percentage of more than 10% in the six-month interval and the presence of desaturation in the baseline 6-minute shuttle walk test, have been used as predictors of survival in IPF patients [31]. In the present study, in patients deceased at the 12-month follow-up, FVC % and DLCO % predicted values were found to be statistically decreased. However, these markers have limitations, such as the need for patient effort and follow-up, and they fail to predict mortality in some cases [31]. Indeed, serum biomarkers could possibly have advantages over physiologic markers, such as ease of sampling and independence from patient effort. 

Regarding the relationship between lung function and biomarker serum levels, our study revealed several results. Specifically, we reported several inverse correlations between the FVC, TLC and DLCO values with serum levels in accordance with previous studies [25]. In addition, in the group of deceased IPF patients at 24 months of follow-up, patients with more than a 10% change in DLCO were found with statistically significant higher concentrations of VEGF-A and CCL18. CCL18 is a CC-chemokine produced by human myeloid-derived cells and is highly expressed in the lung, mainly produced by macrophages [32]. In several previous studies, elevated CCL18 serum levels have been correlated with disease progression, and it has been suggested that CCL18 can predict shorter survival [33,34]. Moreover, Prasse et al. have suggested a cut-off point of serum CCL18 concentration as a routine measurement in the management of IPF patients, although with caution, as CCL18 is increased in other fibrotic lung diseases with prognoses differing from IPF [34]. In addition, concerning gender distribution, in the present study, CCL18 serum levels were found to be statistically increased in male IPF patients. Regarding age, gender and smoking, our results in our cohort agree with previous epidemiological studies. Specifically, the incidence of IPF increases with age, affects males more than females, and smoking is a risk factor [35]. Thus, age, gender, and smoking should also be taken into consideration in the management of this disease. Moreover, the GAP index is a prognostic tool that is commonly used in the management of IPF, which considers three factors: gender, age and pulmonary function (FVC and DLCO) [31]. In our study, the GAP index was related to an increased risk of mortality, both at the 12- and 24-month follow-up, in accordance with previous studies [36]. Also, the GAP index was significantly related to the biomarker combination of KL-6, SP-D and VEGF-A, revealed in our study by ROC curve analysis. In a recent study of 59 IPF subjects, researchers found higher levels of serum lipoproteins to be negatively correlated with the GAP index [37].

As the prognostic impact and mechanisms of comorbidities are not fully understood, we also performed an analysis to evaluate the use of the CCI combined with the GAP index and serum biomarker levels. Most of our patients were classified in groups 2–3 according to the CCI scoring system, regarding category IV of the GAP index. However, we did not find any association with the ROC curve analysis results. A possible explanation is that the patients in our cohort had a relatively low CCI. It has been previously reported that the progression of comorbidities may be pathophysiologically linked to IPF progression, and in terms of that, a Japanese study similarly suggested that these two indexes, GAP and CCI combined, could provide more accurate information for predicting the prognosis in IPF patients [14].

Concerning IL-8, serum levels were found to be inversely correlated to the DLCO values (pred %) in deceased patients. Similar results were reported by Papiris et al., where increased IL-8 levels were related to a higher risk of death in IPF patients [38]. In the present study, not all serum biomarkers were found to be elevated in patients with severe lung decline or disease progression. However, all these molecules are known to participate in the lung fibrosing process and have been shown to be overexpressed in the lung tissue of patients with IPF [9]. 

Several growth factors, such as IGFs (insulin-like-growth factors) and IGFBPs, have been reported to be involved in IPF pathogenesis [9]. In our study regarding IGFBP-1, elevated levels were found to be significantly higher in deceased patients at the 12-month follow-up. Similarly, previous studies have already shown that IGFBPs are increased in IPF [39], and raised levels have also been found in BAL in IPF patients [40]. 

This study has several limitations. In the context of the COVID-19 pandemic, the limitation of having only one blood sample was due to the challenges of recruiting patients, such as restrictions on in-person visits or reduced access to healthcare. Furthermore, the sample size is relatively small, which might underpower our analysis. Another limitation is the absence of additional validation datasets. Indeed, concerning the serum evaluation biomarkers, standardizing the entire procedure, from the blood collection to ELISA measurement, is critical to ensure reliable results. Further research and validation are necessary to confirm serum biomarker usefulness in clinical practice.

Although serum concentration is not an absolute pointer in terms of mortality and might not work in certain patients, the correlation of certain biomarkers to disease progression and mortality has been demonstrated in our study. It is crucial to understand that the usefulness of serum biomarkers for predicting disease severity may vary depending on the specific patient population and other factors, and further research is necessary to confirm their clinical utility [41]. All these biomarkers are not specific to IPF and can be increased in other lung diseases with prognoses that differ from IPF [42]. However, routine measurements of these biomarkers are simple and could provide valuable information in the management of IPF patients. An additional clinical implication of serum biomarkers could be the differentiation of IPF from other lung diseases due to the overlapping clinical and radiological features presented, as previously reported [27].

Regarding antifibrotic treatment, the survival analysis showed no significant differences between nintedanib and pirfenidone treatment in our cohort. Our results indicate that both drugs have similar efficacy in treating IPF. Pirfenidone and nintedanib are the first two FDA-approved therapies for the treatment of idiopathic pulmonary fibrosis [43]. However, it is important to consider additional factors that could impact survival, such as comorbidities, the level of progressive fibrosis and the early initiation of treatment, as these factors could have influenced the treatment response [44]. Furthermore, in our study, no significant associations were found regarding smoking status. A recent study developed a disease progression model to characterize the observed variability in lung function decline and its decrease in decline after antifibrotic treatment [45]. They concluded that smoking status and oxygen use at baseline may affect the treatment effect size. Indeed, identifying prognostic factors that can predict the treatment response in IPF patients could be valuable for designing clinical studies.

## 5. Conclusions

In conclusion, our study revealed that the KL-6, SP-D, VEGF-A, IGFBP-1, ICAM-1 and CCL18 serum levels were significantly elevated in IPF patients with disease progression and death. Furthermore, the majority of serum biomarkers studied were correlated to lung decline. To the best of our knowledge, this is the first report in the Greek population of IPF to combine a broad spectrum of serum biomarkers. Here, we suggest using the combined prognostic value of the GAP index and the KL-6, SP-D and VEGF-A elevated serum levels in IPF patients. In the context of IPF, biomarkers are important for the early detection and early initiation of treatment, differential diagnosis, monitoring disease progression, predicting outcomes for treatment responses and stratifying patients into risk categories. At this point, the value of the combined detection of serum level biomarkers could be used as additional tools in a multidisciplinary approach to IPF, improving the management of vulnerable patients with probable shorter survival and a worse clinical course. It is important to note that the serum biomarker levels alone cannot be used to evaluate the prognosis of IPF, but combined with other tests, such as pulmonary function tests, imaging and genetics, they are more effective. Pulmonary fibrosis biomarkers cannot be considered sufficient, and more research in this area is needed to evaluate and further explore them.

## Figures and Tables

**Table 1:** Clinical characteristics of IPF patients and 24-month evaluation of survival status.

Parameters	All IPF Patients *n* = 72	Alive *n* = 40	Deceased *n* = 32	*p*-Values
Age in years (Mean ± SD)	72 ± 6	71 ± 5	74 ± 6	0.022 *
Sex (male/female)	60 (83%)/12 (17%)	30 (75%)/10 (25%)	30 (94%)/2 (6%)	0.034 *
Smoking Current or ex-smokers/never-smokers	64 (89%)/8 (11%)	34 (85%)/6 (15%)	30 (94%)/2 (6%)	0.240
Pack/years	77.2 ± 171	100 ± 242	55.5 ± 48	0.356
Antifibrotic treatment (nintedanib/pirfenidone)	40 (56%)/32 (44%)	20 (50%)/20 (50%)	20 (63%)/12 (37%)	0.289
LTOT, *n* (%)	22 (30%)	0	22 (69%)	<0.001 *
GAP index, *n* (%) I II III IV	1 (2%) 20 (28%) 31 (43%) 20 (28%)	1 (3%) 14 (35%) 19 (48%) 6 (15%)	0 6 (19%) 12 (38%) 14 (44%)	0.042 *

* p < 0.05 with statistically significant differences between the groups of alive and deceased IPF patients at 24-month follow-up. LTOT: long-term oxygen therapy, GAP: gender–age–physiology.

**Table 2:** Pulmonary function tests at baseline and 12-month follow-up of survival status.

Parameters/Patients	Alive			Deceased		
(%pred), Mean ± SD	Baseline	12 Months	*p*-Values	Baseline	12 Months	*p*-Values
FVC	78.7 ± 14	81.7 ± 13.7	0.007 *	69.8 ± 20.6	71.9 ± 22	0.044 *
TLC	62.1 ± 9.9	61 ± 12	0.201	57.6 ± 12.2	54.8 ± 14	0.068
DLCO	50.3 ± 11.7	52.7 ± 14.3	0.077	41.1 ± 12	36.7 ± 12.1	0.001 *

* *p* < 0.05 with statistically significant differences. FVC: forced vital capacity, TLC: total lung capacity, DLCO: diffusing capacity of the lung for carbon monoxide.

**Table 3:** Serum biomarker levels at 24-month follow-up of survival status.

Biomarkers (Mean ± SD)	All IPF Patients *n* = 72	Alive *n* = 40	Deceased *n* = 32	*p*-Values
KL-6 (U/mL)	648.4 ± 175.8	618 ± 184	684 ± 161	0.117
SP-D (ng/mL)	47.5 ± 24.3	40 ± 25.5	56.3 ± 19.7	0.005 *
IL-8 (pg/mL)	177.4 ±772.8	29.1 ± 51	353.6 ± 1125	0.113
CXCL13 (pg/mL)	8.7 ± 9.5	6.8 ± 8.9	10.7 ± 9.8	0.980
CCL18 (pg/mL)	76.32 ± 42.4	69.5 ± 32	83.9 ± 51	0.164
VEGF-A (pg/mL)	28 ± 39	19.2 ± 6	38.5 ± 56	0.063
IGFBP-1 (pg/mL)	702.2 ± 675	587.1 ± 505	855.6 ± 835	0.100
IGFBP-2 (pg/mL)	237 ± 97	225.5 ± 117	251.4 ± 66	0.261
IGFBP-7(pg/mL)	280 ± 78	277.1 ± 86	285.1 ± 67	0.620
ICAM-1 (pg/mL)	96 ± 41	87 ± 31.9	107.7 ± 47	0.043 *
MPO (pg/mL)	22 ± 25.5	22.8 ± 29.4	21.6 ± 19	0.856
MMP-9 (pg/mL)	18,056 ± 10,952	19,754 ± 12,910	15,793 ± 7209	0.108

* With statistically significant differences between the groups of alive and deceased IPF patients at follow-up.

**Table 4:** Receiver operating characteristic (ROC) curve analyses of KL-6, SP-D and VEGF-A.

Variable	Area	Cut-Off Point	*p*-Values	Sensitivity	Specificity
KL-6 (U/mL)	0.531	607	0.019 *	0.571	0.353
SP-D (ng/mL)	0.750	51.2	0.001 *	0.714	0.176
VEGF-A (pg/mL)	0.500	21.5	0.001 *	0.5	0.239

* *p* < 0.05 with statistical significance.

**Table 5:** GAP index relation to follow-up and ROC curve analysis. Results presented as *n* (%).

IPF Patients/GAP Index	I	II	III	IV	*p*-Values
12-month follow-up					
patients alive	1 (3%)	15 (50%)	10 (33%)	4 (13%)	0.004 *
patients deceased	0	4 (12%)	18 (53%)	12 (35%)	
24-month follow-up					
patients alive	1 (3%)	14 (35%)	19 (48%)	6 (15%)	0.042 *
patients deceased	0	6 (19%)	12 (38%)	14 (44%)	
KL-6, SP-D, VEGF-A combination					
<Cut-off	1 (2%)	17 (40%)	19 (44%)	6 (14%)	0.004 *
>Cut-off	0	3 (10%)	12 (41%)	14 (48%)	

* *p* < 0.05 with statistical significance.

**Table 6:** Charlson Comorbidity Index relation to follow-up and ROC curve analysis. Results presented as *n* (%).

IPF Patients/CCI Groups	0–1	2–3	≥4	*p*-Values
All patients	2 (3%)	48 (67%)	22 (30%)	-
12-month follow-up				
patients alive	0	22 (73%)	8 (27%)	0.265
patients deceased	2 (6%)	20 (59%)	12 (35%)	
24-month follow-up				
patients alive	0	32 (80%)	8 (20%)	0.017 *
patients deceased	2 (6%)	16 (50%)	14 (44%)	
KL-6, SP-D, VEGF-A combination				
<Cut-off	2 (5%)	27 (63%)	14 (32%)	0.421
>Cut-off	0	21 (72%)	8 (28%)	
GAP index				
I	0	1 (100%)	0	0.020 *
II	2 (10%)	7 (35%)	11 (55%)	
III	0	24 (77%)	7 (23%)	
IV	0	16 (80%)	4 (20%)	

* *p* < 0.05 with statistical significance.

## Data Availability

Data are available upon reasonable request.
